# Torque Regulation Is Influenced by the Nature of the Isometric Contraction

**DOI:** 10.3390/s23020726

**Published:** 2023-01-09

**Authors:** Philipp Bauer, João Sá Gomes, João Oliveira, Paulo Santos, Pedro Pezarat-Correia, João R. Vaz

**Affiliations:** 1 Centro Interdisciplinar de Performance Humana (CIPER), Neuromuscular Research Lab, Faculty of Human Kinetics, University of Lisbon, 1495-751 Lisbon, Portugal; 2 Interdisciplinary Research Centre Egas Moniz (CiiEM), Egas Moniz School of Health & Science, 2829-511 Almada, Portugal

**Keywords:** force control, variability, entropy, nonlinear analysis

## Abstract

The present study aimed to investigate the effects of a continuous visual feedback and the isometric contraction nature on the complexity and variability of force. Thirteen healthy and young male adults performed three MVCs followed by three submaximal isometric force tasks at a target force of 40% of their MVC for 30 s, as follows: (i) push isometric task with visual feedback (P_visual_); (ii) hold isometric task with visual feedback (H_visual_); (iii) hold isometric task without visual feedback (H_non-visual_). Force complexity was evaluated through sample entropy (SampEn) of the force output. Force variability was analyzed through the coefficient of variation (CV). Results showed that differences were task-related, with P_visual_ showing higher complexity (i.e., higher SampEn) and decreased variability (i.e., lower CV) when compared with the remaining tasks. Additionally, no significant differences were found between the two hold isometric force tasks (i.e., no influence of visual feedback). Our results are promising as we showed these two isometric tasks to induce different motor control strategies. Furthermore, we demonstrated that visual feedback’s influence is also dependent on the type of isometric task. These findings should motivate researchers and physiologists to shift training paradigms and incorporate different force control evaluation tasks.

## 1. Introduction

The human body obtains information for the modulation of force production from different feedback mechanisms. In isometric tasks, force production is influenced by visual information and the level of force output [1]. The process of visuomotor correction is of particular interest, as it is important for the regulation of force output in relation to a target force, which is called force control [2]. Interestingly, athletes competing in visuomotor demanding disciplines show significantly higher visuomotor abilities (e.g., dynamic acuity, accommodative facility) than non-athletes [3]. Contrarily, in older adults, the visuomotor control mechanism is commonly disrupted and leads to alterations in gait patterns and falls [4]. A greater insight into the influence of visuomotor correction on the modulation of force production is critical to improve force control, and with that, meet specific sports’ demands as well as improve rehabilitation programs.

The amount of visuomotor correction not only depends on the population, but also varies with the amount of visual feedback [5,6]. In fact, the muscle force exerted during a submaximal contraction is steadier (i.e., with less variability) when the gain of visual feedback is increased [7]. However, Baweja et al. [8] have found healthy young adults to show less variable torque output in isometric contractions with less visual feedback due to impaired intramuscular coordination with a significant decrease in motor unit discharge rates and, consequently, decreased agonist activation. Furthermore, studies of bi-manual tasks state that intermuscular coordination is also impaired without visual feedback, which leads to less force control [1,8].

The above-mentioned physiological mechanisms influence motor control, though the literature is inconsistent regarding the influence of visual feedback on the magnitude of force variability (i.e., standard deviation) [5,6,9]. Nonetheless, force error is augmented by removing visual feedback [5,6]. The fewer error corrections that stem from the augmented force error are thought to be associated with a more predictable (i.e., rigid) force time series [10]. On the other hand, a high spatial resolution visual feedback (vs. a low spatial resolution visual feedback) allows for more corrections in the motor process, which leads to the production of a less predictable, less regular, and more complex force output, increasing time to task failure [11].

Besides visual feedback, it is known that joint stability depends on feedback of the somatosensory system [12]. Therefore, it has been shown that the nature of the isometric contraction influences the afferent feedback, which is a source of synaptic input that affects time to task failure [13]. Despite isometric action being characterized by no joint motion, we can distinguish between the following two types of isometric muscle action: pushing (working against a stable resistance) and holding (resisting an impacting force) [14]. Although in both cases no motion is carried out, there are some differences between the two isometric actions, with the most commonly reported distinction being time to task failure (i.e., the duration of maintaining either the position or the force task). Interestingly, isometric pushing leads to a prolonged time to task failure when compared to isometric holding across different muscles, particularly in elbow flexors [15,16] and knee extensors [17]. Following the rationale of Kuznetsov and Riley [11], time to task failure is dependent on different feedback mechanisms. Interestingly, visual feedback and the nature of the isometric contraction may influence not only the magnitude of force variability as has been shown in the literature [5,13,15,16,17], but also the temporal structure of the force output, i.e., force complexity. 

Force or torque complexity, the temporal structure of the fluctuations within the force output (i.e., regularity), has been described as an indirect indicator of the functional capacity of the neuromuscular system [18,19] and provides information about the interactions between components of the neuromuscular system (i.e., motor cortical neurons, spinal motoneurons, muscle fibers, and muscle afferents) that interact to produce complex patterns of force [20,21,22,23]. For example, force complexity has been shown to decrease with aging, with older adults exhibiting a decrease in knee extensors force complexity when compared to younger adults [20]. Moreover, force complexity has also been shown to decrease with pathology, as index finger and thumb force complexity was significantly lower in Parkinson’s disease patients when compared to young and elderly healthy individuals [24]. Additionally, recent research has demonstrated that neuromuscular fatigue leads to a loss of force complexity and reduces the neuromuscular system’s adaptability to external perturbations in both maximal and submaximal intermittent and sustained isometric contractions [18,25]. Nonetheless, little is known about the interactions between visual feedback or the nature of the isometric contraction and torque complexity.

The majority of the studies analyzing force complexity used a push isometric pattern with increased or decreased visual feedback in their experimental design [10,11,26]. Few studies used a non-visual condition in which the participant focuses on a neutral point instead of a line, which is closer to a real-world environment. Those studies which used a non-visual (vs. visual) condition to analyze its effect on force complexity show controversy in their results [27,28,29]. For example, Chow and Stokic [27] found that the withdrawal of visual feedback in submaximal isometric knee extension (10% MVC) in subacute stroke patients in both the paretic and non-paretic leg led to improvement in several force parameters. Notably, the force becomes steadier and its temporal structure more irregular. Interestingly, in control patients, after the withdrawal of visual feedback, force only became temporally more irregular at 50% MVC. Conversely, Li and Wei [28] found that the withdrawal of visual feedback (i.e., when the system is only acting based on somatosensory information) leads to an increased regularity in the temporal structure of force. A particularly interesting study, which takes both of the above-described feedback mechanisms, visual and somatosensory, into consideration, is the one by Skurvydas et al. [29], which states no significant differences regarding torque complexity in knee extension and flexion isometric tasks with or without the presence of visual feedback in both anterior cruciate ligament-deficient legs and healthy legs.

To increase the practical transfer of research to real-world conditions, the present study addresses the lack of torque complexity research regarding studies including a nonvisual feedback condition. Furthermore, considering the importance of somatosensory feedback in those processes, the present study is essential to shed light on the possible influences of different contraction natures on torque complexity. Therefore, the present study aims to investigate the effects of continuous visual feedback and isometric contraction mode on the complexity and variability of force. We hypothesize that continuous visual feedback will lead to higher force regularity, compared to non-visual feedback conditions. Furthermore, considering that the pushing contraction mode is associated to an increased time to task failure, we hypothesize that regularity will be lower during pushing isometric contractions compared to holding. 

## 2. Methods

### 2.1. Participants

Thirteen young and healthy male adults aged 18–35 years were recruited by word of mouth and took part in the present study. All participants, with the exception of one, were right-footed. The inclusion criteria included the absence of severe cardiovascular or pulmonary disease, neurological disorders, lower limb disabilities, and other orthopedic concerns, which might limit force production. Furthermore, participants were asked to not execute heavy leg exercise for 48 h and to avoid drugs, caffeine, tea, or other stimulating substances 12 h before the evaluation. Each subject attended one data collection session and signed a written, informed consent form approved by the institutional review board of the Faculty of Human Kinetics and in conformity with the Declaration of Helsinki. Participants were supervised during the whole evaluation, which assured that risks in terms of health were minimal. 

### 2.2. Experimental Design and Protocol

The Biodex System 3 Pro isokinetic dynamometer (Biodex Medical System 3, Shirley, NY, USA) was used. It was first initialized and calibrated according to the manufacturer’s instructions. Participants were seated with their dominant leg attached to the lever arm of the dynamometer and the position adjusted to ensure that the lateral epicondyle of the femur was in line with the axis of rotation of the lever arm. The relative hip and knee angles were 90°, with full extension being 0°. The lower leg was attached to the lever arm above the malleoli with a Velcro strap. Furthermore, straps were secured firmly across both shoulders and the waist to prevent extraneous movement and the use of the hip extensors during the contractions. All measures were taken at 70 º of knee flexion, and participants were asked not to alter their posture. 

This study required participants to visit the laboratory for one session. Before testing, participants performed a range of submaximal isometric and isokinetic leg extensions to ensure proper familiarization with the testing task and warm-up. Participants were asked to perform three maximal voluntary isometric contractions (MVIC) lasting 3 to 5 s each, with 60 s interval of rest between trials. They were instructed to exert their maximum force as fast as possible with strong verbal encouragement. For the submaximal trials, participants performed three isometric tasks at a target torque of 40% of their MVIC for 30 s, as follows: a push isometric task with visual feedback (P_visual_), a hold isometric task with visual feedback (H_visual_), and a hold isometric task without visual feedback (H_non-visual_). Each submaximal task was performed twice with a 60 s period in between. 

**Figure 1:**
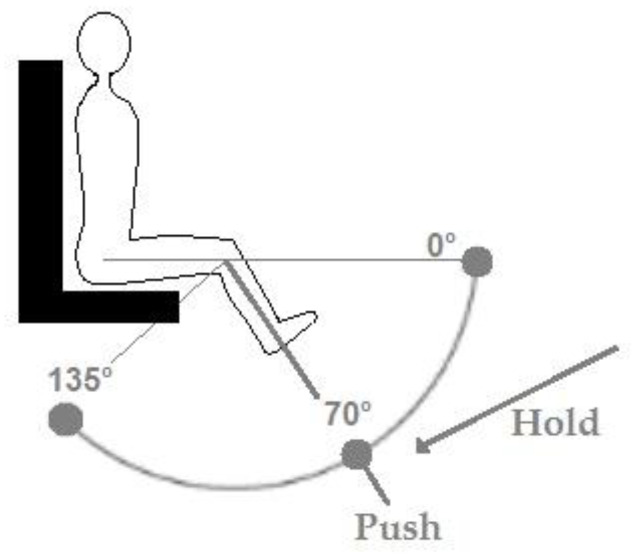
Illustration of force application of the isokinetic dynamometer in the push and hold isometric task.

The visual feedback was provided through a torque target bar displayed on a monitor for the P_visual_ condition, and participants were asked to match their instantaneous torque with the target bar by applying steady force to an immovable resistance (Figure 1). For the H_visual_ condition, visual feedback was delivered by an angular position line, and participants were instructed to align with the testing angle (i.e., 70 º of knee flexion) by sustaining the applied force (Figure 1). In the H_non-visual_ condition, participants were asked to look straight ahead onto a neutral fixed point and, as in the other hold condition, sustain force production at a given angular position of the knee (i.e., 70°). If the testing angle varied by more than 10 degrees from the target angle, the trial was interrupted immediately and repeated. 

### 2.3. Data Analysis

Data were sampled through Biopac MP150 (Biopac Systems Inc., Goleta, CA, USA) interfaced with a personal computer. All signals were sampled at 1000 Hz. Data were collected in Acknowledge (Version 4.1.1. Biopac Systems, Inc.) and further exported to Matlab^®®^ R2018a (The MathWorks, Natick, MA, USA). 

All data were analyzed using code written in Matlab^®®^ R2018a (The MathWorks, Natick, MA, USA). First, the signals were cropped to remove the ascending and descending components of the isometric contractions. Therefore, the analyzed signals only accounted for the time the participant matched the targeted MVIC%. Our power spectral analysis revealed a maximal frequency across all participants of 9 Hz. For further analysis, we used a sampling frequency of 50 Hz, following the recommendation of Stergiou [30] of a sampling frequency five times greater than the highest frequency in the time series of interest. Then, the magnitude of variability, through the coefficient of variation, and temporal structure of variability were calculated. Sample entropy (SampEn) [31] was used to determine the temporal structure of the torque output. Here, SampEn determines the probability that short sequences of data points are repeated throughout a temporal sequence of points. A time series with similar distances between data points would result in a lower SampEn value, while large differences would result in higher SampEn values. Thus, a perfectly repeatable time series has a SampEn value equal to zero, and a perfectly random time series has a SampEn value converging towards infinity [31]. In this study, a pattern length (m) of 2, error tolerance (r) of 0.2, and data length (*n*) of 1500 data points (i.e., 50 Hz × 30 sec) were selected and used in the determination of SampEn values [32]. The reliability of entropy measures was shown to be optimal when these input values are identical for all trials and participants [33].

The coefficient of variation (CV), a common linear measure that translates the amount of variability within the torque signal, was calculated. Mean torque and mean knee joint angle were also calculated to ensure that the targeted torque and position were reached equally across conditions. Here, SampEn, CV, and mean torque were extracted from the exact same previously cropped signal. For statistical purposes, the average of the two trials was used. 

### 2.4. Statistical Analysis

All statistical analysis were carried out using Jamovi (Version 1.6. Sydney, Australia) [34]. Standard descriptive statistics (mean and standard deviation) were used to provide a general overview of the data. A trial-to-trial reliability analysis of the main variable SampEn computing two-way consistency intraclass correlation coefficients (ICCs) and standard errors of the measures ($SEM=SD\times \sqrt{(1}\;-\;ICC)$) for all three conditions was performed prior to the main statistical analysis. All data were tested for normality using the Shapiro–Wilk test. The initial hypothesis was tested using a one-way repeated measures ANOVA (H_non-visual_, H_visual_, P_visual_). Mauchly’s W was used to test sphericity, and Greenhouse–Geisser correction was used when sphericity was not present. If a significant main effect was found, Tukey’s HSD post hoc comparisons were used to further analyze the data. When normality was not present, a non-parametric Friedman’s test was used instead. Additional Durbin–Conover pairwise comparisons were used when a significant main effect was found. Statistical significance was set at *p* < 0.05.

## 3. Results

### 3.1. Reliability Analysis

The ICCs showed 0.899, 0.849, and 0.567, and the SEMs were 0.087, 0.091, and 0.148 for H_non-visual_, H_visual_, and P_visual_, respectively.

### 3.2. Mean Torque

No significant differences were found between conditions (F_(2,24)_ = 1.14, *p* = 0.335, η^2^*_p_* = 0.087).

### 3.3. Torque’s Sample Entropy (SampEn)

**Figure 2:**
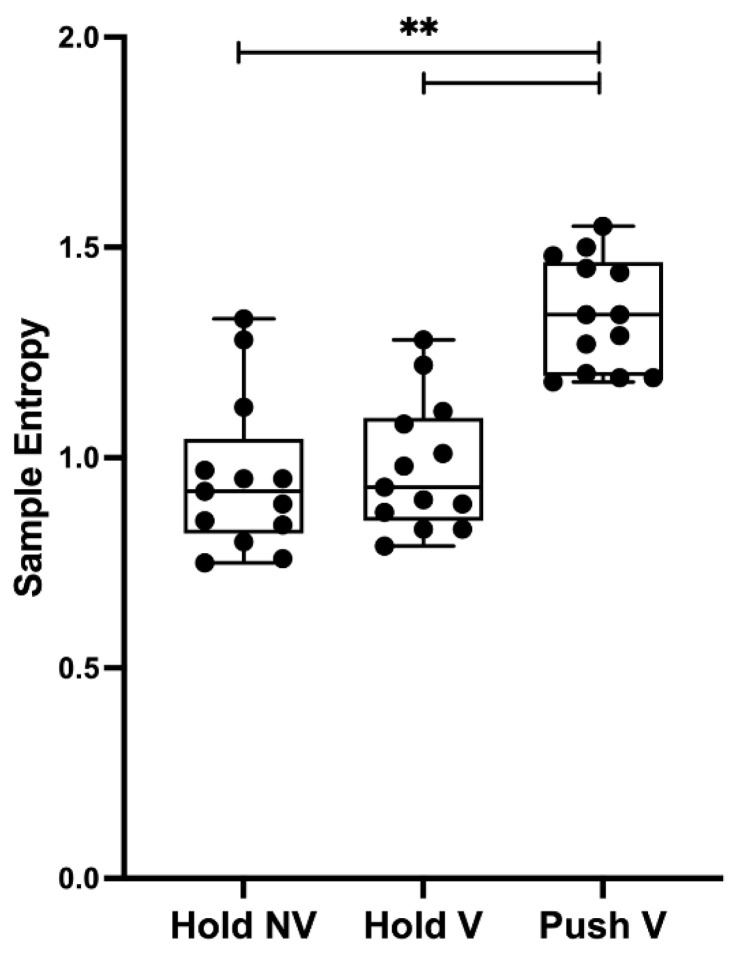
Sample entropy mean values for each condition. Abbreviations are as follows: Hold NV, hold isometric task without visual feedback (H_non-visual_); Hold V, hold isometric task with visual feedback (H_visual_); Push V, push isometric task with visual feedback (P_visual_); ** indicates *p* < 0.001.

A significant effect was found for condition (F_(2,24)_ = 44.90, *p* < 0.001, η^2^ _p_ = 0.789). Post hoc comparisons showed higher SampEn during P_visual_ (1.34 ± 0.13) than H_non-visual_ (0.96 ± 0.18, *p* < 0.001, d = 2.41) and H_visual_ (0.98 ± 0.15, *p* < 0.001, d = 2.51), as can be seen in Figure 2. No significant differences were found between H_visual_ and H_non-visual_ (*p* = 0.651, d = 0.14).

### 3.4. Torque’s Coefficient of Variation (CV)

**Figure 3:**
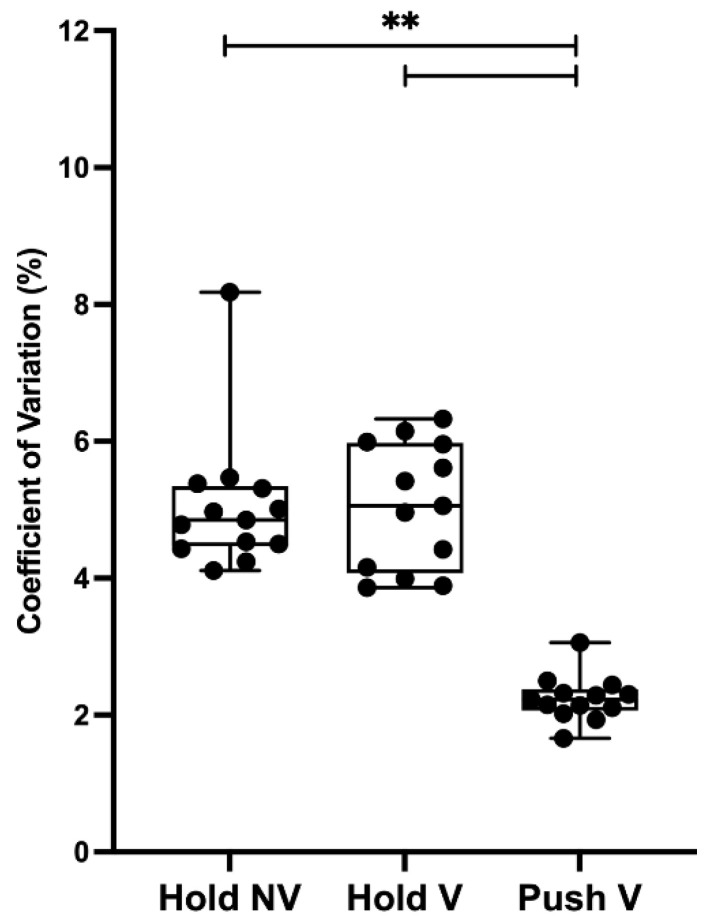
Coefficient of variation mean values for each condition. Abbreviations are as follows: Hold NV, hold isometric task without visual feedback (H_non-visual_); Hold V, hold isometric task with visual feedback (H_visual_); Push V, push isometric task with visual feedback (P_visual_); ** indicates *p* < 0.001.

A significant effect was found for condition (χ^2^_(2,13)_ = 19.5, *p* < 0.001). Pairwise comparisons revealed a greater CV during H_non-visual_ (5.06 ± 1.03) and H_visual_ (5.06 ± 0.91) compared to P_visual_ (2.26 ± 0.36, *p* < 0.001, d = 3.68 and *p* < 0.001, d = 4.09, respectively). No significant differences were found between H_visual_ and H_non-visual_ (*p* = 0.709, d = 0.003), as seen in Figure 3.

### 3.5. Mean Knee Joint Angle

No significant differences were found between conditions (χ^2^_(2,13)_ = 3.23, *p* = 0.199).

## 4. Discussion

This study aimed to investigate the effect of using continuous visual feedback during isometric contraction and how contraction mode would affect torque regulation measures. We hypothesized that continuous visual feedback would lead to greater regularity of torque output compared to the non-visual feedback condition, and that the pushing contraction mode would result in decreased regularity. These hypotheses were partly observed. Specifically, the presence of visual feedback showed no changes; however, pushing mode did exhibit lower regularity (i.e., higher entropy). The trial-to-trial reliability analysis reveals moderate to good reliability of the experimental procedures, with ICCs between 0.567 and 0.899 [35].

Regarding the absence of differences between H_visual_ and H_non-visual_ conditions, a possible explanation for it could be the used contraction intensity of 40% MVC. Chow and Stokic [27] only found differences in torque entropy due to different visual feedback conditions above 50% MVC in healthy adults. The contractions with 10% and 30% MVC showed no differences in torque entropy, as well as the contractions with 20% MVC in the study of Skurvydas et al. [29]. In line with those findings, Pethick et al. [18] state that decreases in torque entropy only occur upon a certain contractile intensity in fatiguing tasks. Nevertheless, it must be taken into consideration that the studies mentioned used knee joint angles of 90° or more, while the present study used 70°, which is considered the optimal angle for force production in knee extension [36]. Pethick et al. [37] showed that decreases in entropy occur only above a certain knee joint angle in fatiguing tasks. Therefore, it is possible that participants in the present study were able to produce a generally more complex force output.

On the other hand, isometric contraction mode led to significant differences. Specifically, the participants showed greater SampEn values during P_visual_ compared to the H_visual_ and H_non-visual_ conditions (i.e., less regularity). Recently, Schaefer and Bittmann [14] showed that a holding elbow extension isometric action at 80% MVC led to a shorter time at an isometric stable position, a shorter isometric *plateau*, and a shorter time to task failure when compared to a similar pushing isometric action. Indeed, a shorter time to task failure is the most commonly found distinction between isometric holding and pushing tasks [15,16,17]. While not a direct measure of our experimental protocol, the longer force endurance of pushing in relation to holding may be associated with different factors which account for such differences.

A possible explanation for our findings could be related to motor control strategies for force production, as they may vary between these two actions since the neuronal control and physiological aspects of a holding isometric muscle action are closer to a lengthening pattern (i.e., an eccentric action) and the underlying mechanisms of a pushing isometric action are similar to a shortening pattern (i.e., a concentric action), without changing the joint angle [14]. Despite being speculative, the findings of Hunter et al. [15] may support this hypothesis, as they found a greater EMG amplitude at exhaustion for pushing when compared to holding isometric actions, a phenomenon that has been demonstrated when comparing concentric and eccentric muscle actions, respectively [38,39]. Furthermore, Grabiner et al. [40] also reported a higher muscle activation when the individual expects a concentric contraction compared to when the individual anticipates an eccentric one.

In terms of complexity and regularity, Hernandez and Camic [41] showed that surface EMG complexity is contraction-type-dependent, as SampEn of the vastus lateralis during maximal effort was higher (i.e., less regular) during concentric contractions in comparison with eccentric contractions. A similar finding was found in the present study regarding torque SampEn, as higher values were observed in pushing contractions (i.e., concentric). Nonetheless, it is not yet known if surface EMG and torque complexity are positively correlated. Directly measuring and analyzing surface EMG through non-linear and linear methods would give us a greater insight regarding the relationship between EMG and torque complexity and the association between holding and pushing isometric actions and eccentric and concentric contractions, respectively. Future studies should also analyze muscular activity to better understand neuromuscular processes related to visual feedback.

The generalizability of the present study might be limited, as the sample consisted only of young male participants. Future studies should include different age groups and female participants to draw out conclusions for different populations. Nonetheless, the findings of the present study indicate that, in healthy young adults, the somatosensory system can compensate a loss of visuomotor correction without any losses of torque complexity. In healthy young individuals, the different feedback mechanisms seem to complement each other in the movement system as a whole. Interestingly, the push and hold isometric contraction patterns appear to induce different motor control strategies. Such findings should motivate health professionals and researchers to shift training paradigms and incorporate different force control evaluation tasks, as we showed that a pushing contraction pattern represents a different motor control strategy when compared to a holding contraction pattern.

## 5. Conclusions

The results of the present study demonstrated that visual feedback did not influence force output’s regularity in both pushing and holding isometric tasks, contrary to our initial hypothesis. We also showed that a pushing contraction pattern, compared to a holding one, results in a less regular force output. These results reveal insights for researchers and clinicians, which should include both contraction patterns in testing and training due to their different motor control strategies.

## Data Availability

The data are available from the corresponding author J.R.V., upon reasonable request.
